# Logistic Organ Dysfunction Score (LODS): A reliable postoperative risk management score also in cardiac surgical patients?

**DOI:** 10.1186/1749-8090-6-110

**Published:** 2011-09-16

**Authors:** Matthias B Heldwein, Akmal MA Badreldin, Fabian Doerr, Thomas Lehmann, Ole Bayer, Torsten Doenst, Khosro Hekmat

**Affiliations:** 1 Department of Cardiothoracic Surgery, Friedrich-Schiller-University of Jena, Erlanger Allee 101, 07747 Jena, Germany; 2 Institute of Medical Statistics, Computer Sciences and Documentation, Friedrich-Schiller-University of Jena, Bachstrasse 18, 07743 Jena, Germany; 3 Department of Anesthesiology and Intensive Care Medicine, Friedrich-Schiller-University of Jena, Erlanger Allee 101, 07747 Jena, Germany; 4 Department of Cardiothoracic Surgery, University of Cologne Kerpener Straße 62, 50937 Cologne, Germany

**Keywords:** Logistic scoring system, Cardiac surgery, Mortality prediction

## Abstract

**Background:**

The original Logistic Organ Dysfunction Sore (LODS) excluded cardiac surgerypatients from its target population, and the suitability of this score in cardiac surgery patients has never been tested. We evaluated the accuracy of the LODS and the usefulness of its daily measurement in cardiac surgery patients. The LODS is not a true logistic scoring system, since it does not use β-coefficients.

**Methods:**

This prospective study included all consecutive adult patients who were admitted tothe intensive care unit (ICU) after cardiac surgery between January 2007 and December 2008. The LODS was calculated daily from the first until the seventh postoperative day. Performance was assessed with Hosmer-Lemeshow (HL) goodness-of-fit test (calibration) and receiver operating characteristic (ROC) curves (discrimination) from ICU admission day until day 7. The outcome measure was ICU mortality.

**Results:**

A total of 2801 patients (29.6% female) with a mean age of 66.4 ± 10.7 years wereincluded. The ICU mortality rate was 5.2% (n = 147). The mean stay on the ICU was 4.3 ± 6.8 days. Calibration of the LODS was good with no significant difference between expected and observed mortality rates on any day (p ≥ 0.05). The initial LODS had an area under the ROC curve (AUC) of 0.81. The AUC was best on ICU day 3 with a value of 0.93, and declined to 0.85 on ICU day 7.

**Conclusions:**

Although the LODS has not previously been validated for cardiac surgerypatients it showed reasonable accuracy in prediction of ICU mortality in patients after cardiac surgery.

## Background

Le Gall et al. initially proposed the Logistic Organ Dysfunction Score (LODS) (Table 1) in 1996 [1]. The authors constructed the score by analyzing the data from 14745 consecutive patients admitted to 137 medical, surgical, or mixed intensive care units (ICUs) in 12 different countries. Burn patients, coronary care patients, and cardiac surgery patients were excluded from the dataset.

**Table 1:** LODS

Organ system	Parameter	5	3	1	0	1	3	5
Neurologic	GCS	3-5	6-8	9-13	14-15	-	-	-

Cardiologic	HR (beats/min)	< 30	-	-	30-139	140	-	-
		
			or			and	or	
		
	SBP (mmHg)	< 40	40-69	70-89	90-239	240-269	≥270	-

Renal	Urea nitrogen (mmol/l) (g/l)	-	-	-	<6 <0.36	6-9.9 0.36-0.59	10-19.9 0.60-1.19	≥20 ≥ 1.20
		
				and	or	or		
		
	Creatinine (μmol/l) (mg/dl)	-	-	-	<106 <1.20	106-140 1.20-1.59	≥141 ≥1.60	-
		
				and		or		
		
	Urine output (l)	<0.5	0.5-0.74	-	0.75-9.99	-	≥10	

Pulmonary	PaO_2 _mmHg/F_i_O_2_ (on MV or CPAP)		<150	≥150	no MV no CPAP	-	-	-
		
	PaO_2 _kPa/FiO_2_	-	<19.9	≥19.9	no IPAP	-	-	-

Hematologic	Leukocytes (× 10 ^9^/l)	-	<1.0	1.0-2.4	2.5-49.9	≥50.0	-	-
		
				or	and			
		
	Platelets (10^9^/l)	-	-	-	<50	≥50		

Hepatic	Bilirubin (μmol/l) (mg/dl)	-	-	-	<34.2 <2.0	≥34.2 ≥2.0	-	-
		
				and	or			
		
	PTtime (secs)	-	-	-	≤3	>3	-	-
		
	above standard (%)			<25	25			

GCS: Glasgow coma scale; SBP: systolic blood pressure; HR: heart rate; PT: prothrombin

In the last few years, some of the general scoring systems have been shown to be valid for use in cardiac surgery patients [2]. Validation of the Sequential Organ Failure Assessment (SOFA) score in 218 cardiac surgical patients has demonstrated that general ICU-scoring systems may be reliable in this patient subgroup without any modification [2]. We, therefore, hypothesized that the LODS might have good predictive power for risk of mortality in cardiac surgical patients.

## Methods

This study involved evaluation of prospectively collected data from all consecutive adult patients admitted to our ICU after cardiac surgery. Patients admitted between January 1^st ^2007 and December 31^st ^2008 were included and the study was approved by the Institutional Review Board of our university (approval no.: 2809-05/10). Only the first admission was considered for patients who were readmitted to the ICU during the study period. Data were collected from the quality control system QIMS 2.0b (University Hospital of Muenster, Germany) and from the intensive care information system COPRA 5.2 (COPRASYSTEM GmbH, Sasbachwalden, Germany), which is interfaced with patient monitors (Philips IntelliVue MP70, Amsterdam, Netherlands), ventilators (Draeger Evita IV, Luebeck, Germany and Hamilton Galileo, Bonaduz, Swizerland), blood gas analyzers (ABL 800Flex Radiometer, Copenhagen, Denmark) and the central laboratories.

The attending physician collected the data and calculated LODS values for the first postoperative week. Two assigned medical clerks validated the data collection daily. A senior consultant performed a second periodical validation. There were no missing data. The LODS was calculated daily using the worst value for each variable per day. Outcome was defined as ICU mortality.

Statistical analyses were performed with SPSS software version 18 (SPSS Inc, Chicago, IL). Graphics were drawn using SigmaPlot software version 11.0 (Systat Software Inc, San Jose, CA, USA). Continuous scale data are presented as mean ± standard deviation (SD) and were analyzed using the two-tailed Student's t-test for independent samples. A p value of < 0.05 was considered as significant. The LODS performance was assessed with the Hosmer-Lemeshow (HL) goodness-of-fit test to insure the absence of a significant discrepancy between predicted and observed mortality. Calibration was considered good when there was a low X^2 ^value and a high p value (> 0.05). Discrimination (ability of a scoring model to differentiate between survival and death) was evaluated with receiver-operating-characteristic (ROC) curves; the area under the curve (AUC) indicates the discriminative ability of the score, i.e., the ability to discriminate survivors from non-survivors. An AUC of 0.5 (a diagonal line) is equivalent to random chance [3], whereas an AUC of 1.0 implies perfect discrimination [4]. The overall correct classification (OCC) (the ratio of the number of correctly predicted survivors and non-survivors to the total number of patients) values of the score were calculated. All statistical analyses were performed from ICU day 1 (n = 2801) (operative day) until the seventh day (n = 338 patients) only, in order to obtain accurate statistical results with sufficient numbers of patients.

## Results

The study included 2801 patients who were admitted to the ICU over the two-year period; 29.6% (n = 830) were female, and the mean age was 66.9 ± 10.7 years (range of 19-89 years). The types of surgical procedure are shown in Table 2. ICU length of stay was 4.3 ± 6.8 days (range 1-189 days, median 2.0 days, 75^th ^percentile 4.0 days) and ICU mortality was 5.2% (n = 147). The preoperative mean additive EuroSCORE was 6.3 ± 3.6 and the mean logistic EuroSCORE was 9.9 ± 12.9.

**Table 2:** Surgical procedures in the study population

Surgery	number	%
CABG	1526	54.5
Isolated valve surgery	635	22.7
Combined CABG and valve surgery	381	13.6
Ascending aorta and aortic arch surgery	60	2.1
Combined ascending aorta and valve surgery	116	4.1
Combined ascending aorta and coronary surgery	5	0.2
Cardiac transplantation	24	0.9
Congenital, cardiac tumors, pulmonary embolectomy, assist devices	54	1.9
**Total**	**2801**	**100**

CABG: Coronary artery bypass grafting

**Figure 1:**
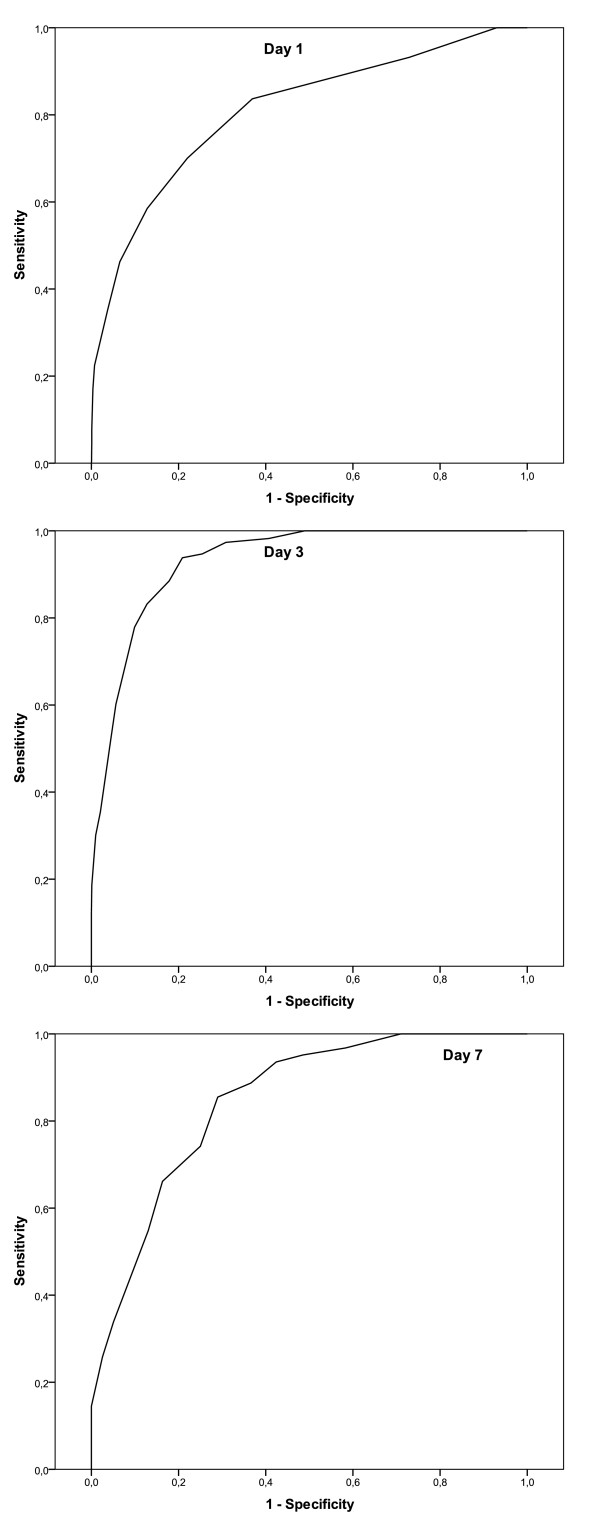
**Receiver Operating Characteristic Curve of LODS on ICU-days 1, 3 and 7**.

There were no significant differences between expected and observed mortality for LODS using the HL-test. The largest AUC was achieved on the third ICU day (AUC = 0.93) and the smallest AUC on the admission day (AUC = 0.81). Figure 1 shows the ROCs of the LODS on days 1, 3, and 7. The OCC was better than 83% on all days with its highest value of 95.7% on the second day. Table 3 summarizes the OCC, calibration and discrimination of LODS from the first ICU day to day 7.

**Table 3:** Summary of overall correct classification (OCC), calibration and discrimination of LODS from ICU-day 1 to day 7

ICU-day	OCC	Calibration		Discrimination	
		Chi^2^	p-value	AUC	95%-CI
1 (n = 2801)	95.3	6.920	0.227	0.810	0.771 - 0.850
2 (n = 2769)	95.7	6.694	0.350	0.913	0.891 - 0.936
3 (n = 1234)	92.2	6.402	0.494	0.930	0.912 - 0.949
4 (n = 815)	90.6	7.928	0.339	0.879	0.844 - 0.914
5 (n = 566)	87.6	5.615	0.690	0.870	0.834 - 0.905
6 (n = 430)	86.5	6.387	0.604	0.847	0.800 - 0.894
7 (n = 338)	83.7	4.663	0.793	0.846	0.799 - 0.893

95%-CI: 95%-Confidence Interval; AUC: Area under the receiver operating characteristic curve; ICU-day: Intensive care unit-day;

## Discussion

The LODS (Table 1) was developed by Le Gall et al. in 1996 [1]. The developmental database for this score was assembled as part of the European/North American Study of Severity Systems (ENAS), which was used to develop the Simplified Acute Physiology Score (SAPS) II for estimating the probability of mortality among ICU patients [5]. Data on 14745 consecutive ICU admissions were collected in 137 medical, surgical, or mixed ICUs in 12 countries to develop and validate the LODS. Eighty percent of the patients in the database were randomly selected for the developmental sample, and the remaining 20% composed the validation sample. As with the development of SAPS II, differences by site were not considered in the development of the system. It is perhaps because cardiac surgical patients were excluded from the original dataset that the LODS has never been tested on this specific patient population. Nevertheless, we demonstrated that the LODS had acceptable accuracy in mortality prediction during the first postoperative week with good calibration on all days, indicating the reliability of LODS in this patient subgroup.

Although the LODS is calculated on the basis of a logistic equation with the statistical technique of multiple logistic regressions, it is not a genuine logistic score because it was transformed into an additive model later in the developmental process. Points are allocated for neurological, cardiovascular, and renal dysfunction and for the pulmonary, hematologic, and hepatic systems and address both the relative severity among organ systems and the degree of severity within an organ system. The total number of points provides an estimated risk of mortality. The additive score correlates with the percentage mortality rate (Table 4). A true logistic score should be calculated according to the well known and established formula used for such a purpose, as does, for example, the logistic EuroSCORE [6], which provides a direct risk of mortality in percentage and not in score points. This formula is: Predicted mortality = exp (β_0 _+ β_1_*x_1_+ β_2_*x_2_+ ...+ β_i_*x_i_)/(1+ exp (β_0 _+ β_1_*x_1_+ β_2_*x_2_+ ...+ β_i_*x_i_)) where β_0 _is the constant of the logistic regression equation and β_i _is the coefficient of a variable. The Xi = 1 when the variable is present and 0 when the variable is absent. Furthermore, a full logistic scoring model is not limited to certain cutoff-points but can be calculated with specific β-coefficients.

**Table 4:** Correlation of the LODS with the percentage mortality rate

LOD-Score	Probability of Mortality in %
0	3.2
1	4.8
2	7.1
3	10.4
4	15.0
5	21.1
6	28.9
7	38.2
8	48.4
9	58.7
10	68.3
11	76.6
12	83.3
13	88.3
14	92.0
15	94.6
16	96.4
17	97.6
18	98.9
19	99.3

During the last twenty years, many scoring systems have been developed for use in ICU patients. These systems have limited applicability in cardiac surgery [7,8] and some, among them the LODS, have excluded cardiac surgery patients from their scope. This group of patients suffers from temporary side effects and pathophysiological effects of the heart-lung-machine,[9,10] which can influence the scores obtained from these systems [11]. These effects include the relatively long mechanical ventilation time needed to stabilize these patients [12,13] and postoperative sedation that limits interpretation of the Glasgow Coma Scale [14]. However, all these factors are temporary and have a limited effect on prognosis. For these reasons, most of the cardiac surgical scoring systems might overestimate the risk of mortality in low risk patients (e.g. isolated coronary artery bypass surgery patients). This is not limited only to postoperative scoring models but is also known in preoperative ones (e.g. EuroSCORE) [15].

Our outcome of interest was ICU mortality [16], rather than in-hospital or 30-day mortality, which are used in the EuroSCORE and other cardiac surgery risk models [17]. Diagnosis and case-mix influence ICU mortality, but in-hospital mortality is influenced by factors beyond the critical care unit and so represents institutional rather than specifically ICU performance [18]. Using ICU-mortality as a short-term outcome measure could be seen as a potential limitation, and much longer periods (60-180 days) have been recommended to capture all of the risks of early death [19]. The main advantage of ICU mortality as a study endpoint is that it reduces any inaccuracies related to variations in ICU discharge patterns among institutions or unrelated deaths (e.g., accidental falls) after discharge.

The LODS was designed to combine measurement of the severity of multiple organ dysfunctions into a single score. The multiple organ dysfunction syndrome is one of the major factors contributing to mortality and prolonged ICU stay [20]. Mortality is strongly related to the number and severity, as well as the duration and type of organ dysfunctions, such that the number of failing organs and the degree of their dysfunction correlates well with an increasing mortality risk [8,21-25]. The LODS may be a tool to identify patients at high risk of developing postoperative severe sepsis. Therefore, daily examination of patients for systemic inflammatory response syndrome (SIRS) criteria is included. The LODS may also be useful to identify the need for early goal-directed therapy [26]. However, our results show that discrimination between survival and death is highest on day three. This represents a shortcoming in this score. Most of the cardiac surgical patients are discharged from ICU on the first or the second postoperative days and only the complex cases remain longer, which makes the mortality prediction with a scoring system much easier. An accurate scoring model should have a high predictive power starting from day one. This fact questions the highest accuracy of LODS on day three; whether it is because of the peak of the organ dysfunction on this day or due to exclusion of the healthiest patients who discharged from the ICU before the third day?

The good calibration of LODS in all days (Table 3) means that this score is reliable in predicting mortality in the whole study group (institutional or national registry level). On the other hand, good discrimination means that this score is useful in predicting mortality on an individual patient level (each patient in ICU). Both functions are necessary for a reliable model [27].

To our knowledge, the operative results, such as postoperative echocardiography and electrocardiography are not considered in any of the present scoring models. These criteria are extremely valuable in cardiac surgical patients and are directly related to outcome. We do recommend considering these data in postoperative risk stratification in cardiac surgical patients.

## Conclusion

Although the LODS has not previously been validated for cardiac surgical patients, it has reasonable accuracy in prediction of ICU mortality in patients after cardiac surgery. The LODS is not a true logistic scoring system, because it does not use β-coefficients.

## Abbreviations

AUC: area under the curve; HL: Hosmer Lemeshow; ICU: intensive care unit; LODS: logistic organ dysfunction scores; OCC: overall correct classification; ROC: receivēr operating characteristic; SAPS: simplified acute physiology score; SOFA: sequential organ failure assessment.

## Competing interests

The authors declare that they have no competing interests.

## Authors' contributions

MH: Conception and design; acquisition, analysis and interpretation of data; drafting the manuscript

AB: substantial contributions to conception and design; revising the manuscript critically for important intellectual content

FD: acquisition and analysis of data; revising the manuscript critically for important intellectual content

TL: substantial contributions to statistical methods and analyses

OB: final approval of the version to be published

TD: final approval of the version to be published

KH: substantial contributions to conception and design; interpretation of data; critically revising the manuscript for important intellectual content

All authors read and approved the final manuscript.
